# The Effects of Selected Hot and Cold Temperament Herbs Based on Iranian Traditional Medicine on Some Metabolic Parameters in Normal Rats 

**Published:** 2014

**Authors:** Shirin Parvinroo, Saleh Zahediasl, Masoumeh Sabetkasaei, Mohammad Kamalinejad, Farzaneh Naghibi

**Affiliations:** a *Department of Traditional Pharmacy, School of Traditional Medicine, Shahid Beheshti University of Medical Sciences, Tehran, Iran.*; b *Endocrine Physiology Research Center, Research Institute for Endocrine Sciences, Shahid Beheshti University of Medical Sciences, Tehran, Iran. *; c *Deptartment of Pharmacology, School of Medicine, Shahid Beheshti University of Medical Sciences, Tehran, Iran.*; d *Department of Pharmacognosy, School of Pharmacy, Shahid Beheshti University of Medical Sciences, Tehran, Iran. *

**Keywords:** Hot and cold temperament herbs, Body weight gain, Food intake, Serum insulin, Serum glucose; Water intake, Urine output

## Abstract

This study was aimed to evaluate the effects of diets containing some hot and cold temperament herb seeds according to Iranian traditional medicine (ITM) on some metabolic parameters in acute (24 h) and sub-acute (7 day) experiments that were performed on rats. For each experiment, effects of diets containing 10% herb seeds in category of hot (anise, fennel, ajowan) and cold (cucumber, watermelon, pumpkin) temperaments were analyzed on body weight gain, food intake, water consumption, urine output, serum glucose (SG) and insulin levels of rats. In the acute experiment, anise or fennel fed groups showed a significant decrease in food intake and there were not any changes in other parameters. The hot temperament groups in comparison with the cold temperament ones showed a significant decrease in food intake and a significant increase in SG level. In the sub-acute experiment, anise and fennel fed groups had a significant decrease in body weight gain on the 4thday. On the 7th day, the anise fed group experienced a significant decrease in body weight gain and a significant increase in SG levels. The groups that were fed hot temperament diets compared to the ones that consumed cold temperament diets showed a significant decrease in body weight gain and food intake rates and a considerable increase in SG levels. Considering the findings of this study, one can conclude that it is possible that hot temperament herbs such as anise and fennel be useful for humans for certain conditions such as weight control.

## Introduction

According to the literature available on ITM, the essential constituents of human and nonhuman beings are just four elements, *i.e*. fire, air, water and soil. Warmness, coldness, wetness and dryness are the basic qualities, each containing a pair of characteristics, fire is warm and dry, air is warm and wet, water is cold and wet, and soil is cold and dry. After integration of these four elements in different quantities, one or two qualities will be dominant in subjects (or objects). This predominant quality (or qualities) is called Mizaj (temperament) (1-3). From this viewpoint, medicines (whether produced from herbs, animals, or minerals), similar to individuals, each have their own specific temperaments. For example lettuce is cold and garlic is hot; when a medicine doesn’t ignite any changes in bodily qualities, it has a moderate temperament; however hot or cold medicines will cause to produce more heat or cold in the body than it already had (2, 4). Temperament is an important and basic concept in ITM for treatment of distemperament diseases and has two categories of 1) related- and 2) non related- to substance (humors) (2, 5); for treatment of these diseases, ancient Iranian physicians, taking into account the incidental temperaments of the patients and the extent of the normal temperament, used to prescribe medicines with opposite temperaments (2). Cold and hot temperament theory not only exists in ITM, but is also accepted in many other civilizations that utilized their own specific traditional medicines such as Indian, European, Arabic, Roman, Greek, and Chinese (6). In fact Hippocrates (460-375 BC), a physician from ancient Greek was the first to mention this theory in his treatise “on the nature of man” (7). It must be noted that the maintenance of the balance between the essential body elements with principal role of warmth and coldness is the main rule of ancient traditional medicine theories (6).

The existing knowledge on the functional mechanisms of medicines based on cold and hot temperaments is not very extensive, and often overlaps in ancient prescriptions; new findings can help us in determining research directions to study these mechanisms. 

According to Jorjani (1042-1136 AC), garlic, caraway and pepper are hot temperament herbs and recommended for weight reduction (5). In 1998 Yuriko Oi and colleagues showed that in rats which were supplemented with garlic, body weight was decreased and thermogenesis was enhanced by increasing both uncoupling protein content (in intercapsular brown adipose tissue) and noradrenaline and adrenaline secretion (8). However, Masjedi, *et al*. study showed that consumption of garlic juice did not alter the body weight of normal rats, but diabetic rats which received the same garlic juice had a significant increase in their body weight compared to untreated diabetic rats (9). In 2010, Dehghani, *et al*. showed that daily use of hydroalcoholic extract of caraway in rats, causes significant weight loss, an increase in T3 and T4 hormones and a decrease in thyrotropin levels (10). More recent findings show that piperine, an active ingredient in black pepper, can induce weight loss with different mechanisms including: increase in thyroid hormone concentrations, bioavailability of many nutrients, gastrointestinal blood flow and secretion of digestive enzymes, and thermogenesis (11). 

In this study, we have investigated the effects of three hot temperament herb seeds including: *Pimpinella anisum *L. (anise), *Foeniculum vulgare *Mill. (fennel) and *Thrachyspermum ammi *(L.) sprague (ajowan) [Syn: *Thrachyspermum copticum *(L.) Link, *Carum copticum *(L.) Benth. and Hook. f.] from the family Apiaceae and three cold temperament herb seeds including: *Cucumis sativus *L. (cucumber), *Citrullus lanatus *(Thunb.) Mansf and Nakai (watermelon) [Syn: Citrullus vulgaris Schard.], *Cucurbita pepo *L*. *(pumpkin) from the family Cucurbitaceae on body weight gain, food intake, urine output, water consumption and non-fasting serum glucose and insulin concentrations in normal rats after periods of 24 h and 7 days of feeding. 

## Experimental

*Food*

The herbs were purchased from a local market in Tehran and authenticated at the department of Traditional Pharmacy, School of Traditional Medicine, Shahid Beheshti University of Medical Sciences. For preparation of the foods, pellets and herbs were powdered by a grinder (cold herbs were sieved to remove seed’s crusts) and mixed thoroughly with some water to form a mixture containing 10% herbs, which was then used for re-pelleting. Pellets were spread on a flat surface for drying at room temperature for 3 days.

*Animals*

Male Wistar rats weighting 200-260 g (5-6 months old) were obtained from the animal house of Shahid Beheshti University of Medical Sciences and were acclimated for 7 days, prior to experiments. Animals were caged individually and maintained in constant temperatures (24 ± 2 °C) and photo schedule (12 h light and 12 h dark) with free access to food and water; they were divided randomly into 7 groups (n = 6) for each test. All experiments were performed in accordance with the Ethics Committee Guidelines for Research on Laboratory Animals of Shahid Beheshti University of Medical Sciences, Tehran, Iran.

*Experimental design*

Two experiments were performed; Protocols 1 and 2 examined the effects of hot and cold temperament herb seeds on food intake, water consumption, urine output, and non-fasting serum glucose and insulin concentration after 24 h, and respectively 7 days. One day before starting the tests, rats were placed in metabolic cages to assess their water consumption, food intake, and urine output (the first day was meant to accustom the rats to new cages). For each of the tests, while the controls received the usual diet, the other groups were fed with prepared diets. In both experiments, animals had free access to water and food. In second experiment, food intake, water consumption and urine output were measured every day and weight was also determined on the 4th and 7th day of the experiment. Food intake and water consumption rates were calculated on daily basis, always at the same time by subtracting the amount of food and water left in the container from the amount of food and water provided the previous day (gm/day/rat and mL/day/rat). Water was measured using a graduated cylinder with 1 ml accuracy. Food and bodies were weighed using an electronic digital scale (HC3000- 3000ˣ0.1g). Daily urine volume was determined by 24 h collection into test tubes that had 0.5 mL calibration between 2 and 15 ml. For reducing the effect of evaporation on urine volume, every 8 h, urine sample of each animal that was in a small container below the metabolic cage was moved to a sealed tube. Upon completion of the experiments, animals were sacrificed under CO2 anesthesia and their blood was collected by the cardiac puncture method. Blood was centrifuged (3000rpm for 10 minutes) and the serum was kept at −70 °C for biochemical analysis. Insulin concentration was determined using the Elisa Rat kit, (Mercodia, Sweden). Glucose was determined using the glucose oxidase peroxidase enzymatic method (Pars Azmoon Co., Iran). Intra- and inter- assay coefficients of variations for glucose and insulin measurements were 2.4%, 8.7% and 5.8%, 10.2% respectively.

*Statistical analysis*

All the data are presented as mean ± SEM. Body weight gain, urine output, water consumption, food intake rate, and serum insulin and glucose concentration were analyzed using a one-way ANOVA (analysis of variance) with Dunnett post- hoc test. Comparisons between the effects observed in the hot temperament groups with those of the cold temperament ones on different metabolic parameters were done by one-way ANOVA. P- values < 0.05 were considered significant.

## Results

*Effects of diets containing hot or cold temperament herb seeds on body weight gain*

The effects of diets containing hot or cold temperament herb seeds on body weight gain in normal rats are illustrated in Table 1. On the 4th day, anise and fennel significantly (P < 0.01) decreased the body weight gain. On the 7th day, anise significantly (P < 0.05) and fennel marginally (P = 0.05) decreased body weight gain. The hot temperament groups compared to the cold temperament ones, showed a significant decrease in body weight gain on the 4th (P < 0.01) and 7th days (P < 0.001) of the study respectively. 

**Table1:** Effects of diets containing hot or cold temperament herb seeds on body weight gain

**body weight gain (g) ** **7**th **day**	**body weight gain (g) ** **4**th **day **	**Groups **
18.9 ± 3.3	14.9 ± 2.2	Control
4.4 ± 5.1*	-2.5 ± 5.2**	*Pimpinella anisum *(anise)
7.1 ± 2.01	-0.5 ± 3.0**	*Foeniculum vulgare *(fennel)
12.0 ± 3.3	9.2 ± 3.1	*Trachyspermum ammi *(ajowan)
22.2 ± 1.7	13.9 ± 3.0	*Cucumis sativus *(cucumber)
17.1 ± 2.1	10.8 ± 2.0	*Citrullus lanatus *(watermelon)
16.4 ± 2.5	7.5 ± 2.5	*Cucurbita pepo *(pumpkin)

Values are mean ± SEM. n=6; * *P*< 0.05, ** *P*< 0.01 denote a significant decrease compared to controls

*Effects of diets containing hot or cold temperament herb seeds on total food intake*

Total food intake was assessed on a 24 h basis in both experiments (Table 2). In the 24 h experiment, anise (P < 0.001) and fennel (P < 0.05) significantly decreased the total food intake. On the 7th day, there were no significant differences in total food intake between the test groups compared to controls. However during the 7 days, fennel significantly (P < 0.05) and anise marginally (P = 0.055) decreased the total food intake. The hot temperament groups compared to the cold temperament ones showed a significant decrease in the total food intake after 24 h (P < 0.001) and during the 7 days (P < 0.05).

**Table2:** Effects of diets containing hot or cold temperament herb seeds on total food intake

**Food intake (g)**			**Control**
**During 7 days in second ** **phase**	**7** ^th^ **day**	**24 h **	
125.3 ± 6.0	17.1 ± 0.7	19.7 ± 0.9	
102.0 ± 6.9	16.3 ± 1.2	13.1 ± 1.0***	*Pimpinella anisum *(anise)
100.6 ± 6.4*	15.0 ± 1.2	15.0 ± 1.7*	*Foeniculum vulgare *(fennel)
116.8 ± 5.9	16.1 ± 0.5	16.4 ± 0.8	Trachyspermum ammi (ajowan)
124.2 ± 6.0	16.9 ± 0.9	19.5 ± 1.0	*Cucumis sativus *(cucumber)
114.8 ± 5.3	17.3 ± 1.1	18.1 ± 0.6	*Citrullus lanatus *(watermelon)
115.9 ± 6.6	16.5 ± 0.8	19.7 ± 1.0	*Cucurbita pepo *(pumpkin)

Values are mean ± SEM. n=12 for 24 h test and n=6 for 7 day test. * *P*< 0.05, ***P< 0.001 denote a significant decrease compared to controls

*Effects of diets containing hot or cold temperament herb seeds on water consumption*

In animals fed with diets containing hot or cold temperament herb seeds, there were no significant differences in the average water intake compared to controls after both the 24 h and the 7 days experiments. Also analysis of total amount of water intake during the 7 days of experiment showed no significant differences between the groups. The hot temperament groups compared to the cold temperament ones showed no significant difference in water consumption during any of the experiments (Table 3).

**Table 3:** Effects of diets containing hot or cold temperament herb seeds on water intake

**Water intake (mL) **			**Control**
**During 7 days in second phase**	**7** ^th^ **day**	**24hr **	
252.1 ± 10.3	33.6 ± 1.4	46.2 ± 3.0	
248.3 ± 12.7	37.1 ± 3.0	37.2 ± 3.3	*Pimpinella anisum *(anise)
240.8 ± 6.1	31.8 ± 1.1	38.6 ± 2.7	Foeniculum vulgare (fennel)
272.0 ± 22.5	33.1 ± 2.2	38.4 ± 1.9	*Trachyspermum ammi *(ajowan)
250.5 ± 12.6	32.1 ± 2.8	45.9 ± 3.5	*Cucumis sativus *(cucumber)
280.1 ± 12.1	35.6 ± 2.5	38.8 ± 1.2	*Citrullus lanatus *(watermelon)
296.6 ± 20.1	34.3 ± 1.9	42.0 ± 2.7	*Cucurbita pepo *(pumpkin)

Values are mean ± SEM. n=12 for 24 h test and n=6 for 7day test. Results show no significant difference between any groups compared to controls in all tests

*Effects of diets containing hot or cold temperament herb seeds on urine output*

There were no significant differences in the urine output of the groups consuming the hot or cold temperament diets compared to controls at any time point of the study; neither did the comparison of hot and cold temperament groups show any significant differences in the amount of urine output (Table 4).

**Table 4:** Effects of diets containing hot or cold temperament herb seeds on urine output

**Urine output (mL)**			**Control**
**During 7 days in second phase**	**7** ^th^ **day**	**24 h **	
53.0 ± 5.6	5.8 ± 0.7	8.7 ± 0.7	
49.5 ± 4.0	6.6 ± 0.4	7.3 ± 0.7	*Pimpinella anisum *(anise)
50.6 ± 2.3	6.5 ± 0.4	7.5 ± 0.7	*Foeniculum vulgare *(fennel)
46.6 ± 4.3	5.8 ± 0.6	7.6 ± 0.6	*Trachyspermum ammi *(ajowan)
41.4 ± 2.1	5.1 ± 0.7	7.2 ± 0.6	*Cucumis sativus *(cucumber)
50.7 ± 3.8	6.5 ± 0.3	8.1 ± 0.7	*Citrullus lanatus *(watermelon)
43.5 ± 3.5	5.2 ± 0.5	8.0 ± 0.9	*Cucurbita pepo *(pumpkin)

Values are mean ± SEM. n=12 for 24 h test and n=6 for 7day test. Results show no significant difference between any groups compared to controls in both tests

*Effects of diets containing hot or cold temperament herb seeds on serum glucose and insulin levels*

Effects of diets containing hot or cold temperament herb seeds on non-fasting serum glucose and insulin levels are shown in Table 5. There were no differences in glucose and insulin levels between the tests groups compared to controls after 24 h; however the hot temperament groups compared to the cold temperament ones showed a significant increase in serum glucose levels (P < 0.05). On the 7th day, anise significantly (P < 0.01) increased serum glucose compared to controls. Also the hot temperament groups, compared to the cold ones, showed a significant increase in serum glucose levels (P < 0.001). There were no significant differences in non fasting insulin levels between test groups compared to controls or in comparison between hot and cold nature groups (Table 5).

**Table 5:** Effects of diets containing hot or cold temperament herb seeds on serum glucose and insulin levels.

**Insulin(μg/dL)** **Day 7**	**Insulin(μg/dL)** **24 h**	**Glucose(mg/dL)** **Day 7**	**Glucose(mg/dL)** **24 h**	**Groups**
1.1 ± 0.1	2.5 ± 0.4	165.9 ± 8.7	169.7 ± 10.6	Control
1.4 ± 0.2	1.4 ± 0.1	213.6 ± 9.8**	232.1 ± 20.1	*Pimpinella anisum *(anise)
1.0 ± 0.1	2.0 ± 0.2	191.7 ± 8.1	260.6 ± 36.8	*Foeniculum vulgare *(fennel)
1.0 ± 0.1	1.5 ± 0.4	177.1 ± 7.3	265.0 ± 23.6	*Trachyspermum ammi *(ajowan)
1.0 ± 0.1	1.8 ± 0.2	164.3 ± 6.0	178.0 ± 11.1	*Cucumis sativus *(cucumber)
1.1 ± 0.0	2.0 ± 0.3	160.9 ± 8.6	216.9 ± 37.6	*Citrullus lanatus *(watermelon)
1.0 ± 0.0	2.5 ± 0.6	175.5 ± 9.8	182.3 ± 33.7	*Cucurbita pepo *(pumpkin)

Values are mean ± SEM. n=6; ** *P*< 0.01 denotes a significant increase compared to controls

## Discussion

This study provides preliminary evidence based on ITM about the effects of diets containing hot or cold temperament herb seeds on several metabolic parameters in normal rats.

*Effects of hot or cold temperament herb on weight gain and food intake*

Our results showed that two of three hot temperament seeds, fennel and anise, were effective in reduction of weight gain and food intake. Others have shown weight (12-16) and appetite (17) reduction properties of selected varieties of hot temperament herbs or their chemical compounds. Various mechanisms have been referred for weight reduction activity of hot temperament herbs such as sympathetic nervous system activation (15,17), increased thermogenesis, activation of AMPK (AMP-activated protein kinase) and decreased expression of multiple genes involved in adipogenesis (14), and increasing energy expenditure and lipid oxidation (16). However the effects of these mechanisms in weight reduction, for hot temperament herb seeds need further examinations. 

Cold temperament seeds did not show any significant effect on weight and food intake in this study. Results which differ from others; The effects of hydroalcoholic and buthanolic extract of cucumber seeds (0.2, 0.4, 0.8 g/Kg) used in normal and streptozotocin-induced diabetic rats for 9 days, showed their effectiveness in controlling the loss of body weight in diabetic rats (18). Ahn *et al*. indicated that administration of 10% watermelon flesh powder for 4-weeks effectively ameliorated streptozotocin-induced weight loss in diabetic ICR mice. Also in this study 1% rind ethanol extract increased their body weight, but not significantly (19). According to current evidence, it seems that doses and the types of herb preparations, animals models (diabetic or non-diabetic) and duration of study can be some of the factors affecting the results. Further studies considering these factors are needed to determine the effects of the cold temperament herb seeds studied here on body weight and food intake.

*Effects of hot or cold temperament herb on water intake and urine output*

Our study found no significant effect for hot or cold temperament seeds on water intake and urine output. Our findings differ from others; the diuretic activity of fennel fruit ethanol extract (500 mg/Kg) dissolved in normal saline (10 mL/Kg) has been shown in mice (20). Beaux *et al*. studied the intraperitoneal usage of fennel hydroalcoholic root extract in saline loaded rats in two experiments. At first when they used the fennel extract they found increased urine volume of 200 mg/Kg after 4, 5, 6, 7 and 8 h, however results didn’t differ after 24 h; in the second experiment, they used lower doses of fennel *(i.e*., 25, 50, 100 and 200 mg/Kg), and only the three highest doses changed the urine volume and its peak was between 4 and 6hrs (21). In the Caceres *et al. *study, administration of 10% whole plant powdered decoction in equivalent doses of 1 g/Kg of fennel by nasogastric catheter indicated an intermediate increasing effect on urine excretion in albino rats after 6 h (22). El Baradei *et al. *studied the daily administration of the fruit extract of fennel for 5 days on SHR and WKY rats, and saw diuretic activity only in the SHR rats (23). Anise oil (0.05%) showed anti diuretic activity in rats but didn’t have any significant effects on water intake during 24 hours (24). Considering the results of mentioned studies and differences in results obtained from our study, these outcomes could be related to the dosage of herb that were used, rat species, route of administration of herbs, different durations of study and the method of diuresis measuring (saline loading vs. non- loading). There is a definite need for more studies, considering these factors.

*Effects of hot or cold temperament herb on serum glucose and insulin*

The hot temperament group of anise increased the glucose concentrations on the 7th day and there was no significant difference in insulin levels between test groups compared to controls. In this study the cold nature seeds didn’t alter the serum glucose and insulin concentrations. These Results differ from the findings of other investigators; Kreydiyyeh *et al. *found that aniseed oil increases glucose absorption in rats’ jejunum through a stimulation of Na+-K+ATPase (24). Methanolic extracts of ajowan (100, 200 mg/Kg) showed some effects lowering the blood glucose in streptozotocin-induced diabetic rats, although not very significant. 

Also an *in-vitro *insulin secretion study of ajowan extract (1, 2 mg/mL) showed its dose dependent insulin secretion in isolated istels of langerhans of streptozotocin induced diabetic rats (25). A hypoglycemic effect was observed in oral administration of fennel essential oil (30 mg/Kg) after 21 days in streptozotocin-induced diabetic rats (26). Minaiyan *et al*. have shown blood glucose reduction using hydroalcoholic and butanolic seed extracts of cucumber (0.2, 0.4, 0.8 g/Kg) in streptozotocin-induced diabetic rats during the sub-acute phase of study, whereas extracts were not effective on reducing blood glucose levels in normal and diabetic rats in acute phase of the study; they proposed beguanid like effects (euglycemic action) of cucumber in diabetic rats (18). The hypoglycemic effects of pumpkin fruit’s pulp and seeds are studied and reported in normal animals, alloxan-induced rats and rabbits and also in type 1 and type 2 diabetic patients (27). Administration of 1% watermelon rind ethanol extract for 4-weeks significantly decreased blood glucose levels and increased serum insulin levels in streptozotocin-induced diabetic ICR mice, but the flesh powder of this herb in 10% doses didn’t show any significant effects on either of parameters (19).

Different doses, animal species diversity, routes of administration, the specific part of the herb that is tested and durations of study are some effective factors which would cause these varied results of the studies mentioned above, indicating the need for further studies to clarify those herbs effects.

By considering glucostatic hypothesis for the regulation of feeding behavior (28) it seems that the hyperglycemia which is caused by anise can probably decrease the food intake and subsequently weight in rats.

In summary, this investigation showed that dietary administration of hot temperament herb seeds compared to cold temperament herb seeds decreased the food intake and weight gain and increased SG levels in normal rats. Considering the findings of this study, one can conclude that it is possible to take advantage from hot temperament herb seeds such as anise and fennel to help humans for conditions such as weight control. 
